# Vegetal and Animal Food Proteins Have a Different Impact in the First Postprandial Hour of Impedance-pH Analysis in Patients with Heartburn

**DOI:** 10.1155/2018/7572430

**Published:** 2018-04-19

**Authors:** Irene Martinucci, Giada Guidi, Edoardo V. Savarino, Marzio Frazzoni, Salvatore Tolone, Leonardo Frazzoni, Lorenzo Fuccio, Lorenzo Bertani, Giorgia Bodini, Linda Ceccarelli, Vincenzo Savarino, Santino Marchi, Nicola de Bortoli

**Affiliations:** ^1^Gastroenterology Unit, Department of Translational Research and New Technologies in Medicine and Surgery, University of Pisa, Pisa, Italy; ^2^Division of Gastroenterology, Department of Surgery, Oncology and Gastroenterology, University of Padua, Padua, Italy; ^3^Gastroenterology Digestive Pathophysiology Unit, Baggiovara Hospital, Modena, Italy; ^4^Division of Surgery, Department of Surgery, University of Campania, Naples, Italy; ^5^Gastroenterology Unit, Department of Internal Medicine, University of Bologna, Bologna, Italy; ^6^Division of Gastroenterology, Department of Internal Medicine (DIMI), University of Genoa, Genoa, Italy

## Abstract

**Background and Aims:**

By means of 24 h impedance-pH monitoring, we aimed to evaluate the effect of two different meals with a bromatological balanced composition: one with a prevailing component of animal proteins and the other with vegetable proteins.

**Patients and Methods:**

We enrolled 165 patients with heartburn and negative endoscopy, who underwent impedance-pH monitoring off therapy. Patients were allocated to receive a Mediterranean diet with a total caloric intake of about 1694 kcal, divided into two meals: one with a prevailing component of animal proteins and the other with vegetable proteins. We evaluated the total reflux number, acid exposure time (AET), and symptom-reflux association with impedance-pH analysis. Moreover, during the first postprandial hour (at lunch and dinner), we evaluated the total reflux number, number of acid and weakly acidic refluxes, AET, and presence of symptoms.

**Results:**

The male/female ratio was 80/85. Mean age was 51.9 ± 12.1 years. Impedance-pH analysis showed that 55/165 patients had pathological AET or a number of refluxes (nonerosive reflux disease (NERD)), 49/165 had normal AET and a number of refluxes but positive symptom-reflux association (hypersensitive esophagus (HE)), and 61/165 had normal AET and a number of refluxes with negative symptom-reflux association (functional heartburn (FH)). The overall first postprandial hour analysis showed a higher total reflux number, acid reflux number, and AET after the animal protein meal than after the vegetable protein meal. Moreover, more symptoms were reported after the animal protein meal. Similar results have been observed in the three different subcategories of patients (NERD, HE, and FH).

**Conclusions:**

Vegetable proteins are associated with a lower number of refluxes, particularly acid refluxes, and with a reduced number of symptoms during the first postprandial hour. This is a pilot study and future investigations are warranted to confirm these results.

## 1. Introduction

Gastroesophageal reflux disease (GERD) is present if the passage of gastric contents back into the esophagus causes either mucosal disease or symptoms [1]. When defined as at least weekly heartburn and/or acid regurgitation, the prevalence in Asia is reported to be less than 5%, whereas that in the Western world generally ranges between 10% and 20% [2–4].

There is evidence that the prevalence of GERD has increased during the past two decades [3, 5]; however, the reason for the rise in GERD and its complications have not been clearly identified. It is likely that an important role is played by the overall change in eating habits: nowadays, the diet in Western countries is mainly characterized by an intake of simple sugars, fats, and animal proteins rather than vegetal ones [6]. Many studies have highlighted that the increase in the prevalence of obesity and GERD are related [7, 8] and recently, it has been demonstrated that a scheduled weight-loss diet may reduce symptoms and PPI consumption in overweight/obese GERD patients [8]. Only few studies evaluated the role of various food components in the genesis of reflux symptoms with contradictory results [9, 10].

The National Institutes of Health and the American College of Gastroenterology recommend that patients with GERD reduce their intakes of total fat, chocolate, alcohol, citrus, tomato products, coffee, tea, and large meals, as well as implement other lifestyle changes, such as stopping smoking and weight reduction. However, based on a low level of evidence, routine global elimination of food that can trigger reflux is not recommended in the treatment of all patients with GERD [11, 12]. To date, there are no data about the role of the different kinds of proteins.

By means of 24 h multichannel intraluminal impedance and pH monitoring (MII-pH), we aimed to evaluate the effect of two different meals with a bromatological balanced composition: one with a prevailing component of animal proteins and the other with vegetable proteins.

## 2. Materials and Methods

Throughout 2017, we enrolled 165 consecutive patients who referred to the outpatient motility laboratories at the University of Pisa for heartburn with or without other GERD-related symptoms. The inclusion criteria were age higher than 18 years and complaints of heartburn with/without regurgitation, at least three times in a week, for 6 months in the previous year. The exclusion criteria were pregnancy (excluded by urine analysis) and/or breast feeding; eating disorders; history of thoracic, esophageal, or gastric surgery; neoplasia; and esophageal motor disorders, outflow obstruction underlying psychiatric illness, or psychiatric therapies. All patients signed the informed consent. The study was designed and carried out in accordance with the Declaration of Helsinki (sixth revision; Seoul, 2008) and was approved by the local institutional review boards.

All patients underwent upper endoscopy to detect erosive esophagitis and/or other esophageal mucosal abnormalities, which has been performed off therapy (proton pump inhibitors, PPIs, or H_2_-receptor antagonists were discontinued at least 20 days prior to endoscopy), within 6 months prior to the initial visit. A distinct investigator completed a detailed patient interview, including a careful review of medical history (with recording of height and weight), current medications, and tobacco and alcohol consumption. All patients completed a detailed questionnaire for GERD diagnosis (GERDQ) [13]. The response to PPI therapy was assessed with a visual analogue scale (VAS) [14].

Then, all the subjects underwent solid-state HRM and MII-pH off therapy (at least a 14-day wash out). Patients were only allowed to take alginates, on an as-needed basis, as rescue therapy for controlling heartburn [15]. The HRM and MII-pH were performed after an overnight fast. HRM was performed to exclude major disorders of peristalsis (i.e., achalasia and EJG outflow obstruction) and to detect the upper border of lower esophageal sphincter (LES). All HRM was performed according to the Italian guidelines of esophageal manometry [16]. The HRM protocol included a 30 sec baseline recording and ten 5 ml water swallows at 20–30 sec intervals in a supine position.

### 2.1. Multichannel Intraluminal Impedance and pH

MII-pH was performed using a polyvinyl catheter (diameter: 2.3 mm), equipped with an antimony pH electrode (Sandhill Scientific Inc., Highlands Ranch, CO, USA). At the end of the recording period, data were edited with a dedicated software program (BioVIEW Analysis, Sandhill Scientific Inc., Highlands Ranch, CO, USA) and analyzed using Microsoft Excel 2000 (Microsoft Inc., CA, USA).

During the MII-pH test, all patients consumed foods and beverages exclusively during three standard meals (lunch at 1.00 p.m., dinner at 8.00 p.m., and breakfast at 8.00 a.m. of the next day).

### 2.2. The One-Day Dietetic Program

Patients were allocated to receive a Mediterranean diet with a total caloric intake of about 1694 kcal, divided into two meals of 847 kcal: one with a prevailing component of animal proteins and the other with vegetable proteins. Both diets were balanced by a caloric point of view and bromatological composition. The one-day dietetic program was calculated by a dietitian (GG) and did not consider the energy requirement of each patient but was calculated for a subject with a BMI ranging between 21 and 22. We created two different dietetic programs: (a) vegetal protein during lunch time and animal protein during dinner time and (b) animal protein during lunch time and vegetal protein during dinner time. The two similar dietetic programs were randomly assigned to the patients who underwent MII-pH. The one-day dietetic program has been detailed in Table 1.

### 2.3. MII-pH Data Analysis

MII-pH tracings were reviewed manually in order to ensure accurate detection and classification of reflux episodes. Meal periods were excluded from the analysis. Impedance and pH data were used to determine the number and type of reflux episodes as well as acid exposure time (AET, reflux percent time) in each patient. Then, a percent time lower than 4.2% with pH < 4, over 24 h, was considered as normal [17]. Acid, weakly acidic, and weakly alkaline refluxes were defined according to the literature [18], and the number of total refluxes was calculated (normal value < 54) [19]. Proximal reflux extents were calculated. Additionally, in a smaller group of patients, similarly distributed among all three subgroups, we evaluated two additional impedance parameters whose role was recently emphasized in terms of diagnostic utility: the PSPW index [20] and mean nocturnal baseline impedance (MNBI) values [21, 22]. The PSPW index, previously described by Frazzoni et al. [23], has been manually calculated, and it is defined as the number of refluxes followed within 30 s by a swallow-induced peristaltic wave divided by the number of total refluxes. This MII-pH parameter shows the efficacy of chemical clearance and was strongly correlated with the presence of esophageal mucosal damage. MNBI values (ohms) were assessed from the same channel (5 cm above the LES) during 30 minutes during the overnight rest, avoiding swallows, refluxes, and pH drops. Indeed, as previously described, short nocturnal time measurements of baseline impedance are reliably representative of long-period measurements [19, 21].

According to the results of MII-pH analysis, all patients were subclassified in three different groups:

- Nonerosive reflux disease (NERD): patients with abnormal acid-exposure time (>4.2%) and/or number of reflux events (>54)
- Hypersensitive esophagus (HE): patients with normal acid exposure and number of reflux events but with a positive correlation between recorded symptoms and reflux events with both indexes (symptom index, SI, positive if >50% and symptom association probability, SAP, positive if >95%)
- Functional heartburn (FH): patients with normal acid exposure time and normal number of reflux events and with no correlation between symptoms and reflux events (SI and SAP negative) including no symptom improvements during previous treatment with PPIs

The random list to suggest an animal protein-based diet during lunch or dinner time was created by means of an Excel easy formula to generate a random list.

We manually calculated the 1 h postprandial AET, number of reflux events, and presence of symptoms to evaluate the effect of the different protein-based (animal or vegetal) food intake. The results of acid exposure in the 1 h postprandial analysis were reported as a percentage of acid exposure (min) up on 60 min of analysis.

### 2.4. Statistical Analysis

Results are reported as mean and standard deviation or absolute frequency and percentage. At univariate analysis, continuous and categorical variables were evaluated with the Student *t*-test and chi-squared test. The Kolmogorov–Smirnov test was used to assess the normality of data. Results were considered statistically significant when *p* value was lower than 0.05. Analyses were performed using SPSS software (version 21; IBM Corp., Armonk, NY, USA).

## 3. Results

We enrolled 165 consecutive patients (85 females), who met the inclusion criteria. Mean age was 51.9 ± 12.1 years, and mean BMI was 23.7 ± 4.1.

All patients reported heartburn (100%), 109/165 (60.1%) reported regurgitation, 38/165 (23%) indicated chest pain, and 26/165 (15.8%) reported extraesophageal symptoms (i.e., cough, pharyngeal globus, and hoarseness). Thirty-four out of 165 (20.6%) patients were used to smoke cigarettes (more than 10 per day), 59/165 (35.8%) patients were used to consume 1-2 alcohol units per day, and 74/165 (44.8%) patients were used to consume, at least, a cup of coffee per day. Moreover, 83/165 (50.3%) showed an improvement lower than 50% on heartburn perception evaluated with VAS scale to the prescribed PPI treatment.

We did not find any manometric abnormalities in the group of selected patients. A manometric confirmed hiatal hernia was found in 63/165 (38.2%) patients. The MII-pH analysis showed that 55/165 (33.3%) were categorized as NERD patients, 49 (29.7%) as HE, and 61/165 (37%) as FH. Patients with NERD had a higher mean AET (6.3 ± 4.8) and a higher number of reflux events (86 ± 33.4) when compared to those with HE (AET 1.9 ± 0.7; reflux events 36.1 ± 9.2) or FH (AET 0.5 ± 0.6; reflux events 24.6 ± 7.9) (*p* < 0.001). Except for hiatal hernia being higher in the NERD group (*p* < 0.001), we did not find any differences in epidemiological characteristics and in voluptuary habits between the three different subgroups. All data are reported in Table 2. Both the baseline impedance value (MNBI) and PSPW index were lower in patients with NERD than in patients with HE and FH (*p* < 0.001). All details regarding the MII-pH were reported in Table 3.

From the analysis of the first postprandial analysis, we observed that 84 patients were randomly assigned to take vegetal proteins during lunch time and animal proteins during dinner time, while 81 patients took animal proteins during lunch time and vegetal during dinner time. We did not observe any differences between patients who consumed animal protein during lunch or dinner time (*p* = 0.503).

Moreover, we observed that when patients consumed animal proteins, they had a three times higher value of acid exposure compared to when they consumed vegetal proteins (AET-1h was, respectively, 3.3 ± 2.7% versus 0.9 ± 1.4%; *p* < 0.005), and similarly, the total number of reflux events was higher after patients consumed animal proteins (total reflux events: 12.4 ± 9.9 versus 6.3 ± 3.9; *p* < 0.0001). Acid reflux events (7.5 ± 4.2 versus 3.3 ± 2.8; *p* < 0.0001) but not nonacid reflux events (5.6 ± 3.5 versus 3.1 ± 2.9; *p* = 0.073) were higher after an animal protein-based diet. A similar result was confirmed for heartburn recorded in the 1 h postprandial analysis, twice more frequently after animal than after vegetal proteins (3.1 ± 1.2 versus 1.4 ± 0.8; *p* < 0.0001). The same results were confirmed when we analyzed patients with NERD, HE, and FH. All details are reported in Table 4.

## 4. Discussion

The prevalence of GERD in the general population is increasing even if the reasons are not completely understood. Changes in eating habits and lifestyle behaviors may play a very important role. It has been hypothesized that eating habits and food choices can play a central role [2]. A correlation between different food combinations and the total number of reflux episodes has been suggested, but rarely was a pathophysiological demonstration found between the occurrence of reflux and different foods. For example, the intake of fatty foods slows gastric emptying and causes a reduction in the basal pressure of the LES through a mechanism probably mediated by cholecystokinin (CCK) that can directly act on the LES, or indirectly, by inhibiting the action of gastrin [24]. For this reason, a low-fat diet has demonstrated a reduction on the onset of GERD symptoms [25]. It has been shown that foods like chocolate and carminatives (i.e., mint) reduce LES basal pressure, favoring the development of GERD symptoms [26].

By the way, lifestyle modifications are the first-line therapy for GERD [11, 27], although adherence to diet is hardly ever optimal [28], both for the long-term cost of the acid-suppressive therapy [29] and for its side effects, such as decreased absorption of dietary calcium and calcium supplement [30], increased risk of hip fractures [31], and an increased risk of clostridium difficile infection [32].

To date, in the literature, there are no studies, which discuss the role of different types of proteins in the pathogenesis of GERD.

The first hour after a meal is considered a moment in which the number of reflux events rise. In line with this assumption, recently, Roman et al. [33] validated the criteria of transient lower sphincter relaxations (TLESRs), by means of high-resolution and impedance manometry in patients evaluated for an hour after a liquid meal containing 600 kcal. Thus, we decided to evaluate the effects of two different meals (with vegetal or animal food protein) with similar caloric amount in the 1 h postprandial analysis to better evaluate the different impact of the two different types of food proteins. Our study showed that during the 1 h postprandial analysis, there was a higher total and acid reflux number of events and a higher AET after the animal protein meal than after the vegetable protein meal. This data was confirmed in all different subgroups of patients with GERD (NERD and HE) and in FH patients. A possible explanation of this finding might be due to an increase of the proximal gastric acid secretion in the postprandial period due to the phenomenon of acid pocket, present in all subjects, not even suffering from GERD [34]. The increased amount of acid in the pocket and the increased acidity of the reflux events might be related to the greater amount of saturated fats in animal protein compared to vegetal ones. In line with this data, an interesting case-control study [35] showed the association between dietary fat and meat intakes in humans and the risk of esophagitis, Barrett's esophagus, and esophageal adenocarcinoma. On the other hand, plant-based fats (e.g., polyunsaturated fat) were not associated with esophagitis and Barrett's esophagus. Moreover, patients in the highest category of meat intake had a higher risk of esophagitis and adenocarcinoma, probably related to monounsaturated and saturated fats. Physiological studies of human volunteers have also shown increased frequency of TLESRs and increased AET with high fat consumption [36, 37]. Boxel et al. [38] showed that heartburn, abdominal discomfort, and nausea increased significantly during lipid infusion in GERD patients, and this finding is to be linked with an enhancement of chylomicron production and secretion that may stimulate release of cholecystokinin, an activator of vagal afferents. Moreover, an important study by Fox et al. [39] evidenced that dietary composition (high-fat diet versus low-fat diet) had effects on esophageal acid exposure (*p* < 0.005) and, above all, symptoms (*p* < 0.001). However, it is necessary to remark that such speculation is about animal products, as a source of animal protein. Future studies are warranted to investigate the direct role of animal proteins.

Our data showed that meat and animal proteins increased both the total amount of acid reflux events and the symptoms. Moreover, some studies regarding hormone release after a meal showed that pork-, beef-, and chicken-derived proteins increased CCK levels in blood after food intake [40, 41]. Even if these studies did not focus their attention on GERD, the CCK levels in the postprandial period seem to increase TLESRs and reduce the basal tone of the lower esophageal sphincter that might easily induce reflux.

It is important to underline that these different types of food (vegetal versus animal proteins) did not modify the 24 h MII-pH analysis of reflux because the MNBI and PSPW index confirmed diagnosis in 100% of the selected patients [42, 43].

The main indication for reflux-related symptoms is focused on avoiding some foods, such as spicy foods, coffee, tobacco, and acidic beverages (orange juice, cola, etc.), from diet. This is the first study that showed that vegetal proteins might reduce the number of reflux events in the first postprandial hour. The limitation of the study is the lack of information regarding the clinical impact of eventual diet modifications.

To conclude, our results showed that vegetable proteins are associated with a lower number of refluxes, particularly acid refluxes, and with a reduced number of symptoms during the first postprandial hour. This is a pilot study and future investigations, such as case control studies, are warranted to confirm these results and their clinical impact.

## Figures and Tables

**Table 1:** The one-day dietetic scheme based on animal food proteins or vegetal food proteins.

		Animal protein-based diet	Vegetal protein-based diet
Carbohydrates, lipids, fruits, and vegetables	*Breakfast*		
	Low-fat milk	150 g	150 g
	Crisp toast	30 g	30 g
	*Lunch/dinner time*		
	Pasta with tomato sauce	70 g	70 g
	Bread	30 g	30 g
	Salad (lettuce)	30 g	30 g
	Extra virgin olive oil	20 g	20 g
	Fruit (apple)	180 g	180 g

Protein content in the diet	Chicken	120 g	N/A
	Baked cod	150 g	N/A
	Mozzarella cheese	140 g	N/A
	Grilled veal steak	130 g	N/A
	Tofu	N/A	320 g
	Soy steak	N/A	55 g
	Grilled seitan	N/A	110 g
	Soy hamburger	N/A	170 g

Nutrition facts	*Total calories*	1694 kcal	1694 kcal
	Carbohydrate % (kcal)	55.5 (940.5)	55.5 (940.5)
	Lipid % (kcal)	25.1 (425.5)	25.1 (425.5)
	Protein % (kcal)	19.4 (327.8)	19.4 (327.8)

**Table 2:** Epidemiological characteristics and voluptuary habits in three different groups of patients.

	NERD (55)	HE (49)	FH (61)	*p*
Male/female	32/23	25/24	23/38	0.089
Mean age ± sd	54.8 ± 13.4	51.7 ± 11.3	49.3 ± 11.7	0.095
Mean BMI ± sd	24.4 ± 3.6	23.8 ± 4.1	22.9 ± 4.6	0.261
Smokers (%)	12 (21.8%)	9 (18.4%)	13 (21.3%)	0.637
Alcohol consumers (%)	23 (41.8%)	18 (36.7%)	18 (29.5%)	0.379
Coffee consumers (%)	30 (54.5%)	23 (46.9%)	21 (34.4%)	0.088
Response to PPI (%)	44 (80%)	34 (69.4%)	0	N/A
Hiatal hernia (%)	42 (76.4%)	20 (40.8%)	1 (1.6%)	**0.001**

BMI: body mass index; PPI: proton pump inhibitors; smokers: habit to smoke cigarettes or cigar; alcohol consumers: ≥2 alcohol units per day; coffee: ≥1 cup of coffee per day.

**Table 3:** MII-pH results in three different subgroups of patients with heartburn.

	NERD (55)	HE (49)	FH (61)	*p*
AET total	6.3 ± 4.8	1.9 ± 0.7	0.5 ± 0.6	**0.001**
AET upright	7.7 ± 4	3.1 ± 1.6	0.8 ± 1.1	**0.001**
AET recumbent	4.8 ± 1.2	0.4 ± 0.6	0.1 ± 0.2	**0.001**
Total reflux events	86 ± 33.4	36 ± 9.2	23.6 ± 7.9	**0.001**
Proximal reflux extension	31.4 ± 7.6	11.8 ± 4.9	9.1 ± 2.3	**0.001**
Acid reflux	58.7 ± 24.3	24.1 ± 6.1	13.1 ± 6.5	**0.001**
Nonacid reflux	27.4 ± 18.9	11.8 ± 6.4	9.5 ± 5.7	**0.001**
SI/SAP positive	70.9% (39/55)	100% (49/49)	0% (0/61)	N/A
MNBI	1047 ± 518	1971 ± 345	3358 ± 762	**0.001**
PSPW index	39.4 ± 5.3	51.7 ± 7.2	71.9 ± 6.8	**0.001**

AET: acid exposure time; SI: symptom index; SAP: symptom association probability; NERD: nonerosive reflux disease; HE: hypersensitive esophagus; FH: functional heartburn.

**Table 4:** 1 h postprandial analysis after animal or vegetal food proteins.

	Postanimal protein	Postvegetal protein	*p*
*Overall analysis*			
PP AET (%)	3.3 ± 2.7	0.9 ± 1.4	**0.005**
PP reflux events (*n*)	12.4 ± 9.9	6.3 ± 3.9	**0.0001**
PP proximal reflux (*n*)	5.2 ± 2.7	1.8 ± 1.3	**0.0001**
PP acid reflux (*n*)	7.5 ± 4.2	3.3 ± 2.8	**0.0001**
PP nonacid reflux (*n*)	5.6 ± 3.5	3.1 ± 2.9	0.073
Symptoms (*n*)	3.1 ± 1.2	1.4 ± 0.8	**0.0001**
*NERD (55 patients)*			
PP AET (%)	6.1 ± 2.7	2.1 ± 0.7	**0.0001**
PP reflux events (*n*)	19.4 ± 9.6	8.1 ± 4.1	**0.0001**
PP proximal reflux (*n*)	6.9 ± 3.2	3.4 ± 1.6	**0.0001**
PP acid reflux (*n*)	11.4 ± 6.2	5.9 ± 2.1	**0.0001**
PP nonacid reflux (*n*)	6.4 ± 2.5	2.8 ± 1.7	**0.0001**
Symptoms (*n*)	4.6 ± 2.3	2.7 ± 1.1	**0.0001**
*HE (49 patients)*			
PP AET (%)	2.9 ± 1.3	0.9 ± 0.3	**0.0001**
PP reflux events (*n*)	8.6 ± 3.1	4.2 ± 1.3	**0.0001**
PP proximal reflux (*n*)	4.8 ± 3.5	1.7 ± 1.5	**0.0001**
PP acid reflux (*n*)	6.3 ± 2.8	3.1 ± 1.9	**0.0001**
PP nonacid reflux (*n*)	3.1 ± 1.4	1.7 ± 0.8	**0.0001**
Symptoms (*n*)	3 ± 1.4	1.6 ± 0.5	**0.0001**
*FH (61 patients)*			
PP AET (%)	0.6 ± 0.3	0.1 ± 0.1	**0.0001**
PP reflux events (*n*)	4.5 ± 1.9	1.8 ± 0.6	**0.0001**
PP proximal reflux (*n*)	2.1 ± 0.7	0.4 ± 0.7	**0.0001**
PP acid reflux (*n*)	3.1 ± 1.1	1 ± 0.4	**0.0001**
PP nonacid reflux (*n*)	1 ± 0.6	0.7 ± 0.3	**0.0001**
Symptoms (*n*)	1.2 ± 0.4	0.3 ± 0.8	**0.005**

PP: postprandial; AET: acid exposure time; NERD: nonerosive reflux disease; HE: hypersensitive esophagus; FH: functional heartburn.
